# Lower Transplacental Antibody Transport for Measles, Mumps, Rubella and Varicella Zoster in Very Preterm Infants

**DOI:** 10.1371/journal.pone.0094714

**Published:** 2014-04-11

**Authors:** Jolice P. van den Berg, Elisabeth A. M. Westerbeek, Gaby P. Smits, Fiona R. M. van der Klis, Guy A. M. Berbers, Ruurd M. van Elburg

**Affiliations:** 1 Department of Paediatrics, Division of Neonatology, VU University Medical Center, Amsterdam, The Netherlands; 2 Laboratory for Infectious Diseases and Screening, National Institute of Public Health and the Environment, Bilthoven, The Netherlands; 3 Centre for Specialised Nutrition, Nutricia Research, Utrecht, The Netherlands; Public Health England, United Kingdom

## Abstract

**Background:**

Maternal antibodies, transported over the placenta during pregnancy, contribute to the protection of infants from infectious diseases during the first months of life. In term infants, this protection does not last until the first recommended measles-mumps-rubella vaccination at 14 months in the Netherlands, while these viruses still circulate. The aim of the study was to investigate the antibody concentration against measles, mumps, rubella and varicella (MMRV) in mothers and preterm infants or healthy term infants at birth.

**Methods:**

Antibody concentrations specific for MMRV were measured in cord blood samples from preterm (gestational age <32 weeks and/or birth weight <1500 g) and term infants, and matched maternal serum samples, using a fluorescent bead-based multiplex immune-assay.

**Results:**

Due to lower placental transfer ratios of antibodies against MMRV in 96 preterm infants (range 0.75–0.87) compared to 42 term infants (range 1.39–1.65), the preterm infants showed 1.7–2.5 times lower geometric mean concentrations at birth compared to term infants. Maternal antibody concentration is the most important determinant of infant antibody concentration against MMRV.

**Conclusions:**

Preterm infants benefit to a lesser extent from maternal antibodies against measles, mumps, rubella and varicella than term infants, posing them even earlier at risk for infectious diseases caused by these still circulating viruses.

## Introduction

Newborn infants, especially preterm infants, have an immature immune system, which is not capable of protecting them actively against vaccine preventable infections such as measles, mumps, rubella and varicella. Maternal Immunoglobulin G (IgG) is transported across the placenta (transplacental transport) by an active, receptor mediated process during pregnancy thereby protecting term infants against infections.[1] In general, higher IgG concentrations are associated with longer protection. Previous studies showed that the degree of transplacental transport of IgG is dependent on the duration of the gestation.[2]–[6] In the first trimester, a small amount of IgG is transported to the fetus.[7], [8] While the fetal IgG is approximately 10% of the maternal concentration at 17–22 weeks of gestation, it increases to 50% at 28–32 weeks of gestation as determined by chordocentesis.[1], [9] The increase of fetal IgG concentrations between 29 and 41 weeks of gestation is twice that at 17 to 28 weeks of gestation.[8]

Besides gestational age, maternal IgG antibody level and IgG subclass are important predictors of the neonatal IgG antibody level, as previously shown for Pertussis, Diphtheria, Tetanus, *Haemophilus influenzae* type B, *N meningitidis* C and varicella zoster.[6], [10], [11] In term infants, the IgG antibody concentration at birth is usually higher than the maternal IgG antibody concentration, especially for IgG1 vaccine antibodies.[6], [8], [12]

In the Netherlands, vaccination of preterm infants is recommended according to the same immunization schedule as term infants, regardless of prematurity. In the Dutch National Immunization Programme (NIP), measles, mumps and rubella (MMR) vaccines are administered at 14 months of age and a booster dose at 9 years of age. The vast majority of the mothers in this study have followed the regular Dutch NIP including MMR. Rubella vaccination (at the age of 11 years) and measles vaccination (at the age of 14 months) were implemented respectively in 1974 and 1976. The MMR-combination vaccine was implemented in 1987 with a catch-up campaign.[13] Any antibodies against mumps or measles in mothers born before 1975 and antibodies against rubella in mothers born before 1963, must therefore be naturally acquired. It is known that infants born to vaccinated mothers are likely to have lower transplacentally acquired maternal antibodies at birth than infants of mothers with naturally acquired antibodies.[14]–[17] Waaijenborg et al.[18] recently showed that the duration of protection of maternal antibodies for measles, mumps and rubella (3.3, 2.7 and 3.9 months respectively) ended well before the age of the first MMR vaccination at 14 months.

An epidemic of measles is on-going in the orthodox Protestant communities of the Netherlands (Dutch Bible belt) since 2013 and an outbreak of rubella occurred within a primary school for children from these orthodox reformed groups.[19] Complication rates of measles are highest in <5 year olds and in particular otitis media, pneumonia, corneal ulcer and subacute sclerosing panencephalitis are most common in <2 year olds.[20]

We hypothesize that IgG antibody concentrations at birth against measles, mumps, rubella and varicella are lower in preterm infants than in term infants, posing them at earlier risk for these infectious diseases. Therefore, the aim of this study was to investigate the concentration of antibodies against measles, mumps, rubella and varicella in mothers and their preterm infants with gestational age (GA) <32 weeks and/or birth weight (BW) <1500 g, and in mothers and their healthy term infants. In addition, follow up of protection in preterm infants was investigated by measuring the concentration of antibodies against measles, mumps, rubella and varicella at 5 months of age.

## Materials and Methods

### Study population

Preterm infants with a GA <32 weeks and/or BW <1500 g and their mothers were eligible for this study. Term infants (GA >37 weeks) and their mothers served as controls. All infants were born at the VU University Medical Center, Amsterdam, the Netherlands.

### Ethics statement

This study was part of the CARROT study, registered as ISRCTN16211826 [21] and conducted according to the guidelines laid down in the Declaration of Helsinki and the medical ethical review board of VU University Medical Center approved all procedures involving human patients. Written informed consent was obtained from all mothers for maternal and cord blood and all parents for follow up.

### Laboratory analysis

We obtained cord blood from the placenta directly after delivery. If cord blood could not be obtained, blood from the infants was obtained within 48 hours after delivery. Maternal blood was obtained by venous sampling between two days before and two days after delivery. Follow up samples of preterm infants were collected 4–6 weeks after the primary series of DTaP-IPV-Hib and pneumococcal vaccinations. Serum samples were centrifuged and stored at −80°C until analysis. Antibody concentrations in the serum samples were analyzed as previously described by Smits et al.[22] In short, serum samples were tested for antibodies against measles, mumps, rubella and varicella zoster virus (VZV) using a fluorescent bead-based multiplex immuno assay (MIA) (Luminex xMAP technology). Serum samples were diluted 1/200 and 1/4000 in phosphate buffered saline (PBS) containing 0.1% Tween 20 and 3% bovine serum albumin (BSA). An in-house standard that was calibrated against the international standards for measles, rubella and VZV, controls and blanks were included on each plate. Antibody concentrations were obtained by interpolation of the mean fluorescent intensity (MFI) in the reference curve using a logistic-5PL regression type and expressed in international units per ml (IU/ml) or RIVM units/ml (mumps). Analysis was performed with a Bio-Plex 200 in combination with Bio-Plex manager software (Bio-Rad Laboratories, Hercules, CA).

### Data analysis

GA, BW and maternal age are described as median and range. For statistical analysis, antibody concentrations below the lower limit of quantitation were assigned as half the lower limit of quantitation (0.002 IU/ml for measles, 0.2 RU/ml for mumps 0.02 IU/ml for rubella and 0.01 mIU/ml for varicella).[18]) All IgG antibody concentrations were expressed as geometric mean concentrations (GMCs) with 95% confidence intervals (CI). Student's *t* test was used to compare the specific antibody concentrations between preterm and term infant serum samples after natural logarithmic transformation. Placental transfer of IgG antibodies to the vaccine components was defined as the ratio between each individual paired infant to maternal serum sample. In addition, the overall ratio for each antigen was defined as the geometric mean of the individual ratios.

International assigned protective concentrations were used to determine the percentage of mothers and infants with protective IgG concentrations (anti-measles ≥0.2 IU/ml [23], [24], anti-rubella ≥10 IU/ml [25]). Protective concentrations for anti-mumps and anti-varicella antibodies are not internationally assigned, but locally assigned cut-off values of ≥45 RU/ml and ≥0.26 IU/ml, respectively, were used as previously described.[18]

A Pearson correlation was performed to determine the maternal IgG antibody concentration in relation to infant IgG antibody concentration. A multiple linear regression analysis was performed to determine the influence of GA, BW, maternal IgG antibody titer and maternal age on infant GMCs and transplacental transport ratio. For all statistical analyses, a two-sided p value of <0·05 was considered significant. SPSS 21·0 (SPSS Inc., Chicago, IL, USA) was used for data analysis.

## Results

### Study participants

Between April 2007 and December 2008, 96 preterm infants and 42 term infants and their mothers were included in this study.

In the preterm group, GA was 29.7 weeks (25.0–32.7), BW was 1235 g (500–2240) and 26/96 (27%) infants had a GA <28 weeks. In the term group, GA was 38.9 weeks (36.7–41.3) and BW 3421 g (2370–5070). Maternal age was lower in the preterm group than in the term group: 32.0 years (19–41) and 34.2 years (21–43), respectively (p = 0.009). In the preterm group, 57% was born by vaginal delivery and 43% by Caesarean section whereas in the term group 29% was born by vaginal delivery and 71% by Caesarean section. In the preterm group 60/97 infants (63%) received prenatal corticosteroids and none of the infants received infant immunoglobulin. In the preterm group, 81 cord serum samples, 10 arterial serum samples, 3 capillary serum samples and 2 venous serum samples were collected and in the term group 42 cord serum samples were collected. At 5 months blood samples of 88 preterm infants were collected; 80 venous samples and 8 capillary samples.

### Seroprevalence of measles, mumps, rubella and varicella vaccine-specific IgG in maternal and infant serum samples

**Figure 1:**
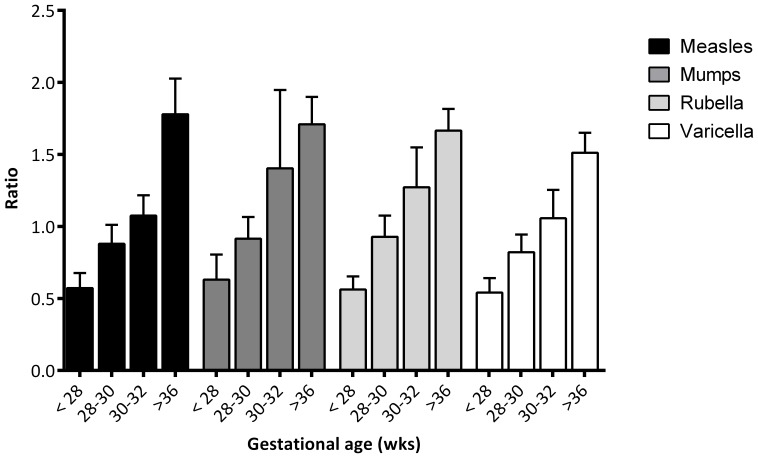
Transplacental transport ratio of measles, mumps, rubella and varicella with 95% confidence intervals.

GMC's, 95% CI, and ranges of measles, mumps, rubella and varicella antibodies in maternal and infant serum samples are summarized in table 1. Placental transfer ratio for the antibodies specific against protein vaccines of measles, mumps, rubella and varicella was lower in preterm infants (0.81, 0.84, 0.87, 0.75 respectively) than in term infants (1.65, 1.53, 1.62, 1.39 respectively, all p<0.01) (table 1). The median placental transfer ratio of all antibodies against the protein vaccines is 0.82 in preterm infants and 1.55 in term infants. Placental IgG transfer of all antibodies was lower in preterm infants <28 weeks than in preterm infants >28 weeks (0.53 vs 0.92, p<0.001). Active transport of maternal antibodies to measles, mumps and rubella was already seen in preterm infants with GA of 30 weeks (fig 1).

**Table 1:** Number of samples tested, geometric mean concentration (GMCs) and transplacental transport ratios of antibodies to measles, mumps, rubella and varicella zoster in preterm and term infants.

	GMCs (95% CI) [range] and transplacental transport ratio				
	N Cord serum		N Maternal serum		Ratio
Measles (IU/ml) preterm	97	0.6 (0.4–0.7) [0.01–30]*	89	0.8 (0.6–0.94) [0.01–12.68]	0.81*
Measles (IU/ml) term	42	1.4 (0.9–2.0) [0.04–19]	39	0.89 (0.60–1.34) [0.03–10.44]	1.65
Mumps (RU/ml) preterm	97	110 (87–139) [2–1511]*	89	136 (107–173) [3–1536]	0.84*
Mumps (RU/ml) term	42	199 (157–253) [24–1055]	39	130 (98–170) [19–814]	1.53
Rubella (IU/ml) preterm	97	38 (30–49) [0–337]^†^	89	49 (38–64) [0–406]	0.87*
Rubella (IU/ml) term	42	66 (42–102) [0–545]	39	48 (32–71) [0–291]	1.62
Varicella zoster (IU/ml) preterm	97	0.63 (0.52–0.77)[0.01–6.71]*	89	0.88 (0.73–1.06) [0.05–5.11]	0.75*
Varicella zoster (IU/ml) term	42	1.52 (1.22–1.89) [0.51–11.05]	39	1.11 (0.86–1.43) [0.27–11.23]	1.39

**NOTE**. Maternal serum samples were obtained from mothers between 2 days before and after delivery and cord serum samples were obtained from umbilical cords. CI, confidence intervals; IU, international unit; RU, RIVM unit; IgG, immunoglobulin G; *p<0.01, ^†^p<0.05.

### Determinants of infant GMC of the vaccine components

**Figure 2:**
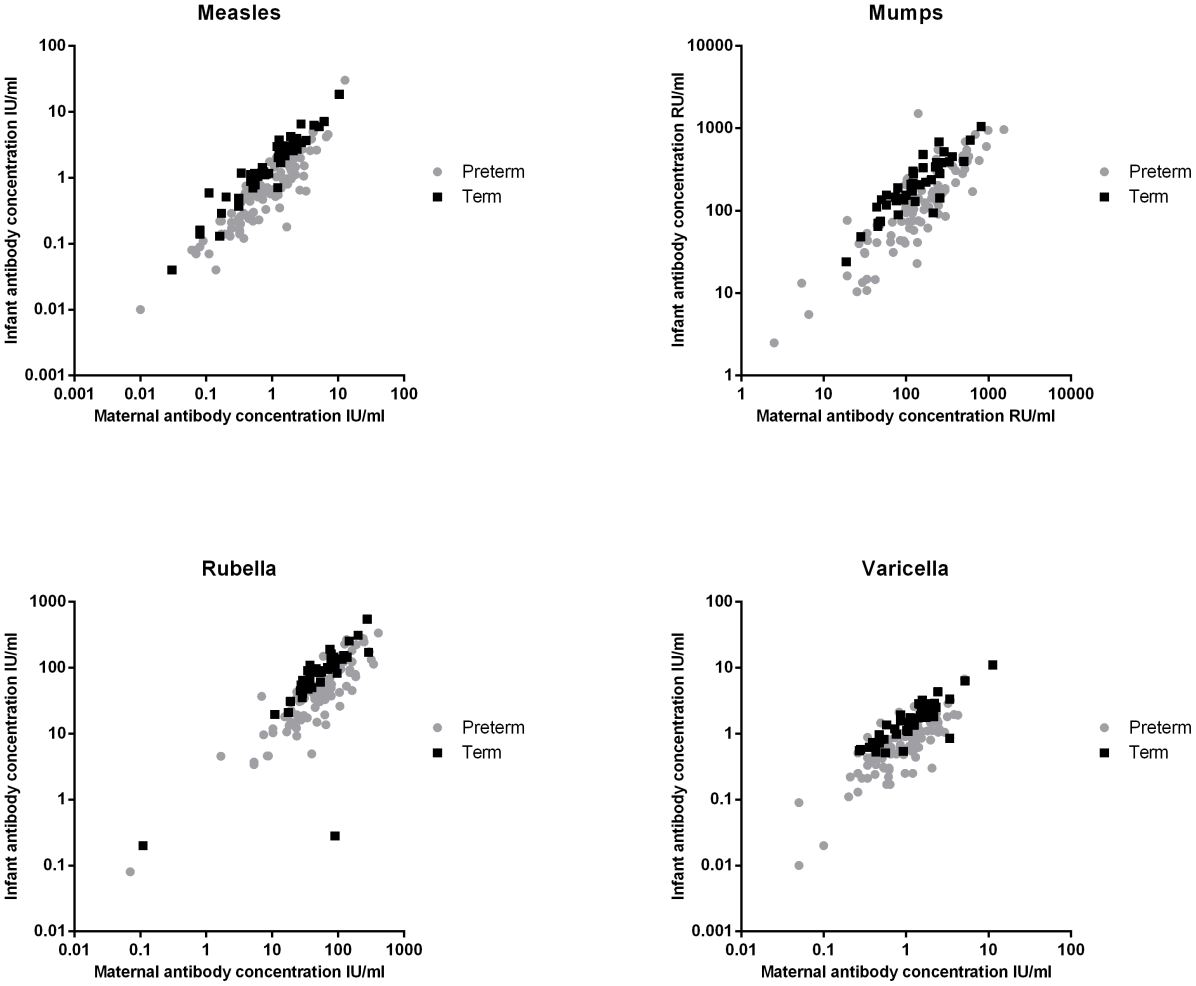
Individual maternal-to cord blood antibody concentrations of measles (IU/ml), mumps (RU/ml), rubella (IU/ml) and varicella (IU/ml).

A strong correlation between maternal and infant antibody concentration was found for all measured antibodies against all vaccine components in both preterm infants (R^2^: 0.68–0.83) and term infants (R^2^: 0.52–0.91) (fig 2). Using a multiple linear regression model, the neonatal GMCs of antibodies against all vaccine components was predominantly determined by the maternal GMC, both in preterm (β: 0.69–0.82, all *P*<0.001) and term (β: 0.80–1.0, all *P*<0.001) infants. In preterm infants, the influence of GA on GMC of MMRV antibodies was less strong compared to the influence of maternal GMC (β: 0.08–0.32, *P*<0.05, except for measles). BW did not influence the GMC against the antibodies specific for the 4 vaccines (all *P*>0.05). In the group of term infants, GA and BW had no influence on the GMCs of antibodies against the vaccine components (*P*>0.05), except for measles (BW β: −0.18, *P* = 0.002). Maternal age did not influence the GMC of any vaccine component in both preterm and term infants (all *P*>0.05).

### Determinants of placental transfer ratio

The placental transfer ratio for antibodies specific for MMRV was strongly influenced by GA (β: 0.69–0.98, all *P*≤0.01) and less by BW (β: −0.41– −0.34, all *P*>0.05, except for measles, *P* = 0.03).

### Protective antibody concentrations in maternal and infant serum samples

Protective antibody concentrations at birth are shown in table 2. Percentages of infants with protection antibody concentrations were lower in preterm infants for mumps and varicella (*P*<0.01), but not significantly lower for measles (*P*:0.10) and rubella (*P*:0.50), compared to term infants. After 5 months 99% of preterm infants had no protective maternal antibody levels for any of the 4 measured antibodies.

**Table 2:** Percentage of protected preterm and term infants and their mothers at birth to *measles, mumps, rubella, varicella zoster*.

		Protective concentrations (%)			
		Preterm		Term	
	Cut off level protection	Infant n: 96	Mother n: 89	Infant n: 42	Mother n: 39
Measles	≥0.2 *IU/ml*	80	86	91	87
Mumps	≥*45* *RU/ml*	78	83	98	92
Rubella	≥*10* *IU/ml*	91	92	95	95
Varicella zoster	≥*0.26* *IU/ml*	84	91	100	100

IU, international unit; RU, RIVM unit.

## Discussion

This study demonstrates lower transplacental transport of IgG antibodies against measles, mumps, rubella and varicella in the vulnerable group of preterm infants with GA <32 weeks compared to term infants with GA>36 weeks, resulting in lower antibody levels in preterm infants at birth.

Lower IgG concentrations indicate shorter duration of protection. Previously, Waaijenborg et al [18] showed that in general the Dutch population lose protective concentrations of maternal antibodies for measles at approximately 3.3 months, for mumps at 2.7 months, for rubella at 3.9 months and for varicella at 3.4 months, well before the first vaccination. Although percentages of levels of protection were not very different between preterm (78–91%) and term infants at birth (91–100%), the GMC's of preterm infants were significantly lower compared to term infants (2.5-fold for measles, 1.8-fold for mumps, 1.7-fold for rubella and 2.4-fold for varicella). This implies that preterm infants will lose their protective maternal antibodies approximately one month earlier than term infants. At 5 months, 99% of the preterm infants did not have protection to measles, mumps or rubella and therefore will be unprotected until the first vaccination, at 14 months in the Netherlands.

In the Netherlands, the previous measles epidemic was in ‘99-’00, with more than 3200 cases and 3 measles-related deaths.[16] The last documented outbreak was in 2008 [26] and currently a measles epidemic is on-going in the orthodox Protestant communities.[19], [27] In the past 10 years, there were still outbreaks of measles with measles-related deaths all over Europe.[28], [29] Infants (younger than 1 year) have the highest age-specific measles-incidence in Europe and are vulnerable for measles-related complications like otitis media, pneumonia, corneal ulcer and subacute sclerosering panencephalitis.[29], [30] Although the goal of the WHO was to eliminate measles in 2010, in 2013 infants are still infected with measles in Europe.[31], [32] Increasing herd immunity by enhanced administration of MMR vaccination is therefore important for elimination of measles in Europe.

As well as outbreaks and epidemics of measles, outbreaks of rubella and mumps also still occur in the Netherlands. In the last decade, outbreaks of mumps increased affecting especially vaccinated student populations, but fortunately not yet involving infants <12 months. During the inclusion period of this study in 2007–2008, zero and seven cases respectively of mumps were diagnosed in the Netherlands.[33] Since 2009, an ongoing outbreak of mumps occured in the student population.[34], [35] For rubella, the last epidemic episode occurred in 2004 in the Netherlands. In 2007 and 2008, 4 and 2 cases respectively of rubella were diagnosed. In 2013, there was an outbreak of rubella which appears to be restricted to one primary school for children from the orthodox reformed groups.[19] So all 3 viruses in the MMR-vaccine are still circulating in the Netherlands, posing a risk of infection to the vulnerable group of non-vaccinated infants and making close monitoring necessary.

Varicella vaccination is not recommended in the Netherlands, but it is in other countries.[36] Varicella infection occurs at a younger age in the Netherlands compared to other countries. In the Netherlands, more than 95% of the children of 6 years of age were found seropositive for varicella, which might explain the low disease burden due to varicella.[37]

Vaccination induces lower antibody levels compared to natural infection,[38], [39] and due to high vaccine coverage, antibody levels will in general not be boosted by exposure to the wild-type virus.[40] In the coming years, almost all women of child bearing age will be vaccinated during childhood and therefore have lower antibody levels against measles, mumps and rubella.[41], [42] In our study, 63% of the mothers of preterm infants were born before 1975 and therefore not vaccinated against mumps or measles, but were vaccinated against rubella. This virus however, was still circulating as only girls were vaccinated until 1987. This allows natural boosting of antibody concentration in addition to vaccination.[43] In the coming years, the period of protection against MMRV in vulnerable preterm infants will decrease further because of lower maternal antibody concentrations. As the elimination of the vaccine preventable diseases is not yet achieved in Europe, adequate protection of vulnerable preterm infants during the first year of life is necessary. To reduce the risk of preterm infants to measles, mumps and rubella, early vaccination at 6–9 months is an option. However, seroconversion is shown to be lower after early vaccination due to the presence of maternal antibodies and immaturity of the immune system.[44] Circulating maternal antibodies inhibit vaccine responses in infants by formation of immune complexes and epitope specific masking of B cell determinants. However T cell priming is not prevented by maternal antibodies.[45] Inhibition by maternal antibodies cannot be the only reason for low seroconversion, as both preterm and term infants lose their antibodies well before vaccination, even if the vaccination is given at 6–9 months instead of 12–14 months. Low seroconversion is also caused by immaturity of the immune system[46], [47] and maybe more important for an adequate response to MMR vaccinations in preterm infants administered at less than 12 months of age, compared to term infants. In case of an outbreak of measles vaccination at 6 months, in addition to the vaccination at 14 months, might be an option. During outbreaks in the US, infants between 6 and 12 months of age received additional vaccination, followed by the routine immunization schedule, at 12 months and 4–6 years.[48] During the last outbreak of measles in the Netherlands, an additional dose of MMR vaccine was also offered.[49] Evidence for MMR vaccination at 6 months in preterm infants is scarce, but Ichikawa et al showed that early immunization at 6 months of age was protective against measles in 17 preterm infants.[50] If there is no direct risk of contact with the viruses, longer protection by maternal antibodies in combination with the first vaccination after 12 months (as previously recommended) may increase protection. In our study, maternal antibody level is the major predictor of the antibody level in the infant. However, vaccination of mothers during pregnancy is contra-indicated as the MMR vaccine contains live attenuated viruses.[51], [52] Therefore, the only option to increase maternal antibodies is to vaccinate women of child-bearing age before pregnancy, but little is known about the effect of MMR vaccination in adults.

Some limitations of the study need to be addressed. First, it was not always possible to obtain cord blood from all infants in our study. In 15 out of 96 cases, other blood samples (venous or arterial) were used for IgG antibody measurements. However, this difference did not influence the results of the study.[53] Second, the follow-up blood sampling was chosen at 5 months because this was 4–6 weeks after the primary series of DTaP-IPV-Hib vaccinations. Therefore, we could not investigate the decay rates of MMRV antibodies in our cohort, but had to use the decay rates from the study of Waaijenborg et al., performed in the same country with a comparable population.[18] In the term group a high rate of Caesarean sections were seen (71%). The mothers of the relatively small group of term infants all delivered in the VU University medical center, a level III hospital. Generally, a university hospital has a high rate of tertiary referral deliveries with comorbidity and therefore higher numbers of infants delivered by Caesarean section. This will not influence the results of the study, as IgG levels were comparable with a previous study. [18]

In conclusion, transplacental transport of IgG is significantly lower in preterm infants than in term infants. Therefore, preterm infants will be at risk earlier and for a longer period than term infants for measles, mumps and rubella, which are still circulating in our population.

## References

[1] Malek A, Sager R, Kuhn P, Nicolaides KH, Schneider H (1996) Evolution of maternofetal transport of immunoglobulins during human pregnancy. Am J Reprod Immunol 36: 248–255. 8955500 10.1111/j.1600-0897.1996.tb00172.x

[2] Leineweber B, Grote V, Schaad UB, Heininger U (2004) Transplacentally acquired immunoglobulin G antibodies against measles, mumps, rubella and varicella-zoster virus in preterm and full term newborns. Pediatr Infect Dis J 23: 361–363. 15071296 10.1097/00006454-200404000-00019

[3] Linder N, Tallen-Gozani E, German B, Duvdevani P, Ferber A, et al (2004) Placental transfer of measles antibodies: effect of gestational age and maternal vaccination status. Vaccine 22: 1509–1514. 15063576 10.1016/j.vaccine.2003.10.009

[4] Linder N, Waintraub I, Smetana Z, Barzilai A, Lubin D, et al (2000) Placental transfer and decay of varicella-zoster virus antibodies in preterm infants. J Pediatr 137: 85–89. 10891827 10.1067/mpd.2000.106902

[5] van den Berg JP, Westerbeek EA, van der Klis FR, Berbers GA, van Elburg RM (2011) Transplacental transport of IgG antibodies to preterm infants: a review of the literature. Early Hum Dev 87: 67–72. 21123010 10.1016/j.earlhumdev.2010.11.003

[6] van den Berg JP, Westerbeek EA, Berbers GA, van Gageldonk PG, van der Klis FR, et al (2010) Transplacental transport of IgG antibodies specific for pertussis, diphtheria, tetanus, haemophilus influenzae type b, and Neisseria meningitidis serogroup C is lower in preterm compared with term infants. Pediatr Infect Dis J 29: 801–805. 20803841 10.1097/inf.0b013e3181dc4f77

[7] Jauniaux E, Jurkovic D, Gulbis B, Liesnard C, Lees C, et al (1995) Materno-fetal immunoglobulin transfer and passive immunity during the first trimester of human pregnancy. Hum Reprod 10: 3297–3300. 8822462 10.1093/oxfordjournals.humrep.a135906

[8] Simister NE (2003) Placental transport of immunoglobulin G. Vaccine. 21: 3365–3369. 10.1016/s0264-410x(03)00334-7 12850341

[9] Malek A (2003) Ex vivo human placenta models: transport of immunoglobulin G and its subclasses. Vaccine 21: 3362–3364. 12850340 10.1016/s0264-410x(03)00333-5

[10] van Der Zwet WC, Vandenbroucke-Grauls CM, van Elburg RM, Cranendonk A, Zaaijer HL (2002) Neonatal antibody titers against varicella-zoster virus in relation to gestational age, birth weight, and maternal titer. Pediatrics 109: 79–85. 11773545 10.1542/peds.109.1.79

[11] de Voer RM, van der Klis FR, Nooitgedagt JE, Versteegh FG, van Huisseling JC, et al (2009) Seroprevalence and placental transportation of maternal antibodies specific for Neisseria meningitidis serogroup C, Haemophilus influenzae type B, diphtheria, tetanus, and pertussis. Clin Infect Dis 49: 58–64. 19480581 10.1086/599347

[12] Pitcher-Wilmott RW, Hindocha P, Wood CB (1980) The placental transfer of IgG subclasses in human pregnancy. Clin Exp Immunol 41: 303–308. 7438556 PMC1537014

[13] Blume S, Tump J (2010) Evidence and policymaking: The introduction of MMR vaccine in the Netherlands. Soc Sci Med 71: 1049–1055. 20667640 10.1016/j.socscimed.2010.06.023 PMC2941041

[14] Zanetta RA, Amaku M, Azevedo RS, Zanetta DM, Burattini MN, et al (2001) Optimal age for vaccination against measles in the State of Sao Paulo, Brazil, taking into account the mother's serostatus. Vaccine 20: 226–234. 11567768 10.1016/s0264-410x(01)00267-5

[15] van den Hof S, Berbers GA, de Melker HE, Conyn-van Spaendonck MA (1999) Sero-epidemiology of measles antibodies in the Netherlands, a cross-sectional study in a national sample and in communities with low vaccine coverage. Vaccine 18: 931–940. 10580207 10.1016/s0264-410x(99)00348-5

[16] van den Hof S, Conyn-van Spaendonck MA, van Steenbergen JE (2002) Measles epidemic in the Netherlands, 1999–2000. J Infect Dis 186: 1483–1486. 12404165 10.1086/344894

[17] Hartter HK, Oyedele OI, Dietz K, Kreis S, Hoffman JP, et al (2000) Placental transfer and decay of maternally acquired antimeasles antibodies in Nigerian children. Pediatr Infect Dis J 19: 635–641. 10917222 10.1097/00006454-200007000-00010

[18] Waaijenborg S, Hahne SJ, Mollema L, Smits GP, Berbers GA, et al (2013) Waning of Maternal Antibodies Against Measles, Mumps, Rubella, and Varicella in Communities With Contrasting Vaccination Coverage. J Infect Dis 208: 10–16. 23661802 10.1093/infdis/jit143 PMC4043230

[19] Mollema L, Hahné S (2013) Measle surveillance report [Mazelensurveillanceoverzicht]. RIVM.

[20] McLean HQ, Fiebelkorn AP, Temte JL, Wallace GS (2013) Prevention of measles, rubella, congenital rubella syndrome, and mumps, 2013: summary recommendations of the Advisory Committee on Immunization Practices (ACIP). MMWR Recomm Rep 62: 1–34. 23760231

[21] Westerbeek EA, van Elburg RM, van den Berg A, van den Berg J, Twisk JW, et al (2008) Design of a randomised controlled trial on immune effects of acidic and neutral oligosaccharides in the nutrition of preterm infants: carrot study. BMC Pediatr 8: 46. 18947426 10.1186/1471-2431-8-46 PMC2579424

[22] Smits GP, van Gageldonk PG, Schouls LM, van der Klis FR, Berbers GA (2012) Development of a bead-based multiplex immunoassay for simultaneous quantitative detection of IgG serum antibodies against measles, mumps, rubella, and varicella-zoster virus. Clin Vaccine Immunol 19: 396–400. 22237896 10.1128/CVI.05537-11 PMC3294611

[23] Chen RT, Markowitz LE, Albrecht P, Stewart JA, Mofenson LM, et al (1990) Measles antibody: reevaluation of protective titers. J Infect Dis 162: 1036–1042. 2230231 10.1093/infdis/162.5.1036

[24] Christenson B, Bottiger M (1990) Methods for screening the naturally acquired and vaccine-induced immunity to the measles virus. Biologicals 18: 207–211. 2257133 10.1016/1045-1056(90)90008-n

[25] Skendzel LP (1996) Rubella immunity. Defining the level of protective antibody. Am J Clin Pathol 106: 170–174. 8712168 10.1093/ajcp/106.2.170

[26] Hahne S, te Wierik MJ, Mollema L, van Velzen E, de Coster E, et al (2010) Measles outbreak, the Netherlands, 2008. Emerg Infect Dis 16: 567–569. 20202450 10.3201/eid1602.090114 PMC3322001

[27] Swart E, Knol M (2013) Measles and rubella surveillance report [Mazelen en rubella surveillance overzicht].

[28] Leuridan E, Sabbe M, Van Damme P (2012) Measles outbreak in Europe: susceptibility of infants too young to be immunized. Vaccine 30: 5905–5913. 22841972 10.1016/j.vaccine.2012.07.035

[29] Muscat M, Bang H, Wohlfahrt J, Glismann S, Molbak K (2009) Measles in Europe: an epidemiological assessment. Lancet 373: 383–389. 19131097 10.1016/S0140-6736(08)61849-8

[30] Strebel P PM, Dayan G, Halsey N. (2008) Measles vaccines. In: O. W. Plotkin S, Offit P, editor editors. Vaccines.Philadelphia: Saunders. pp. 354.

[31] World Health Organization (2005) Eliminating measles and rubella and prevention congenital rubella infection. WHO European region strategic plan 2005–2010. WHO.

[32] European Centre for Disease Prevention and Control (2013) Measles and rubella monitoring. Stockholm: ECDC.

[33] European Centre for Disease Prevention and Control (2010) Annual Epidemiological Report on Communicable Diseases in Europe 2010. Stockholm: ECDC. 22114980

[34] Greenland K, Whelan J, Fanoy E, Borgert M, Hulshof K, et al (2012) Mumps outbreak among vaccinated university students associated with a large party, the Netherlands, 2010. Vaccine 30: 4676–4680. 22579874 10.1016/j.vaccine.2012.04.083

[35] Whelan J, van Binnendijk R, Greenland K, Fanoy E, Khargi M, et al. (2012) Ongoing mumps outbreak in a student population with high vaccination coverage, Netherlands, 2010. Euro Surveill 15.. 10.2807/ese.15.17.19554-en 20460086

[36] Nardone A, de Ory F, Carton M, Cohen D, van Damme P, et al (2007) The comparative sero-epidemiology of varicella zoster virus in 11 countries in the European region. Vaccine 25: 7866–7872. 17919788 10.1016/j.vaccine.2007.07.036

[37] van Lier A, Smits G, Mollema L, Waaijenborg S, Berbers G, et al (2013) Varicella zoster virus infection occurs at a relatively young age in The Netherlands. Vaccine 31: 5127–5133. 23973248 10.1016/j.vaccine.2013.08.029

[38] Janaszek W, Slusarczyk J (2003) Immunity against measles in populations of women and infants in Poland. Vaccine 21: 2948–2953. 12798638 10.1016/s0264-410x(03)00113-0

[39] Kacica MA, Venezia RA, Miller J, Hughes PA, Lepow ML (1995) Measles antibodies in women and infants in the vaccine era. J Med Virol 45: 227–229. 7775944 10.1002/jmv.1890450220

[40] Ohsaki M, Tsutsumi H, Takeuchi R, Kuniya Y, Chiba S (1999) Reduced passive measles immunity in infants of mothers who have not been exposed to measles outbreaks. Scandinavian journal of infectious diseases 31: 17–19. 10381212 10.1080/00365549950161826

[41] Pabst HF, Spady DW, Marusyk RG, Carson MM, Chui LW, et al (1992) Reduced measles immunity in infants in a well-vaccinated population. Pediatr Infect Dis J 11: 525–529. 1528642 10.1097/00006454-199207000-00004

[42] Maldonado YA, Lawrence EC, DeHovitz R, Hartzell H, Albrecht P (1995) Early loss of passive measles antibody in infants of mothers with vaccine-induced immunity. Pediatrics 96: 447–450. 7651776

[43] de Haas R, van den Hof S, Berbers GA, de Melker HE, Conyn-van Spaendonck MA (1999) Prevalence of antibodies against rubella virus in The Netherlands 9 years after changing from selective to mass vaccination. Epidemiol Infect 123: 263–270. 10579446 10.1017/s0950268899002939 PMC2810758

[44] Borras E, Urbiztondo L, Costa J, Batalla J, Torner N, et al (2012) Measles antibodies and response to vaccination in children aged less than 14 months: implications for age of vaccination. Epidemiol Infect 140: 1599–1606. 22074684 10.1017/S0950268811002184

[45] Siegrist CA (2003) Mechanisms by which maternal antibodies influence infant vaccine responses: review of hypotheses and definition of main determinants. Vaccine 21: 3406–3412. 12850349 10.1016/s0264-410x(03)00342-6

[46] Klinge J, Lugauer S, Korn K, Heininger U, Stehr K (2000) Comparison of immunogenicity and reactogenicity of a measles, mumps and rubella (MMR) vaccine in German children vaccinated at 9–11, 12–14 or 15–17 months of age. Vaccine 18: 3134–3140. 10856793 10.1016/s0264-410x(00)00096-7

[47] Redd SC, King GE, Heath JL, Forghani B, Bellini WJ, et al (2004) Comparison of vaccination with measles-mumps-rubella vaccine at 9, 12, and 15 months of age. J Infect Dis 189 Suppl 1S116–122. 15106100 10.1086/378691

[48] Arciuolo RJM, Tamara R. Brantley, MPH, Mekete M. Asfaw, Rachel R. Jablonski, Jie Fu, PhD, Francesca R. Giancotti, PhD, Jennifer B. Rosen, MD, (2013) Notes from the Field: Measles Outbreak Among Members of a Religious Community — Brooklyn, New York, March–June 2013. Morbidity and Mortality Weekly Report. PMC4585576 24025758

[49] National Institute of Public Health and the Environment (2011) LCI guideline Measles (morbilli) [LCI richtlijn Mazelen (morbilli)].

[50] Ichikawa T, Tsuji A, Fujino M, Kusano R, Sugiyama R, et al (2013) Effect of early measles vaccination (AIK-C strain) for preterm infants. Pediatr Int 55: 163–168. 23379893 10.1111/ped.12064

[51] McLean HQ PFA, Temte JL, Wallace GS (2013) Prevention of Measles, Rubella, Congenital Rubella Syndrome, and Mumps, 2013: Summary Recommendations of the Advisory Committee on Immunization Practices (ACIP). MMWR Recomm Rep 62: 1–34. 23760231

[52] Practice Committee of American Society for Reproductive Medicine (2008) Vaccination guidelines for female infertility patients. Fertil Steril 90: S169–171. 19007619 10.1016/j.fertnstert.2008.08.056

[53] Pinsky NA, Loepfe TR, Jacobson RM, Vierkant RA, Poland GA (2003) Comparison of fingerstick versus venipuncture for antibody testing of measles and rubella. Scand J Infect Dis 35: 107–109. 12693560 10.1080/0036554021000027008

